# FAS—A Fully Actuated Segment for Tendon-Driven Continuum Robots

**DOI:** 10.3389/frobt.2022.873446

**Published:** 2022-04-26

**Authors:** Reinhard M. Grassmann, Priyanka Rao, Quentin Peyron, Jessica Burgner-Kahrs

**Affiliations:** Continuum Robotics Laboratory, Department of Mathematical and Computational Sciences, University of Toronto Mississauga, Mississauga, ON, Canada

**Keywords:** continuum robot manipulator, soft manipulator, design, degrees of freedom, helical tendon routing, tendon actuation, follow-the-leader deployment, position redundancy

## Abstract

We propose a segment design that combines two distinct characteristics of tendon-driven continuum robots, i.e. variable length and non-straight tendon routing, into a single segment by enabling rotation of its backbone. As a result, this segment can vary its helical tendon routing and has four degrees-of-freedom, while maintaining a small-scale design with an overall outer diameter of 7 mm thanks to an extrinsic actuation principle. In simulation and on prototypes, we observe improved motion capabilities, as evidenced by position redundancy and follow-the-leader deployment along spatially tortuous paths. To demonstrate the latter on a physical prototype, a simple, yet effective area-based error measure for follow-the-leader deployment is proposed to evaluate the performance. Furthermore, we derive a static model which is used to underpin the observed motion capabilities. In summary, our segment design extends previous designs with minimal hardware overhead, while either archiving similar accuracy in position errors and planar follow-the-leader deployment, or exhibiting superior motion capabilities due to position redundancy and spatial follow-the-leader deployment.

## 1 Introduction

**FIGURE 1:**
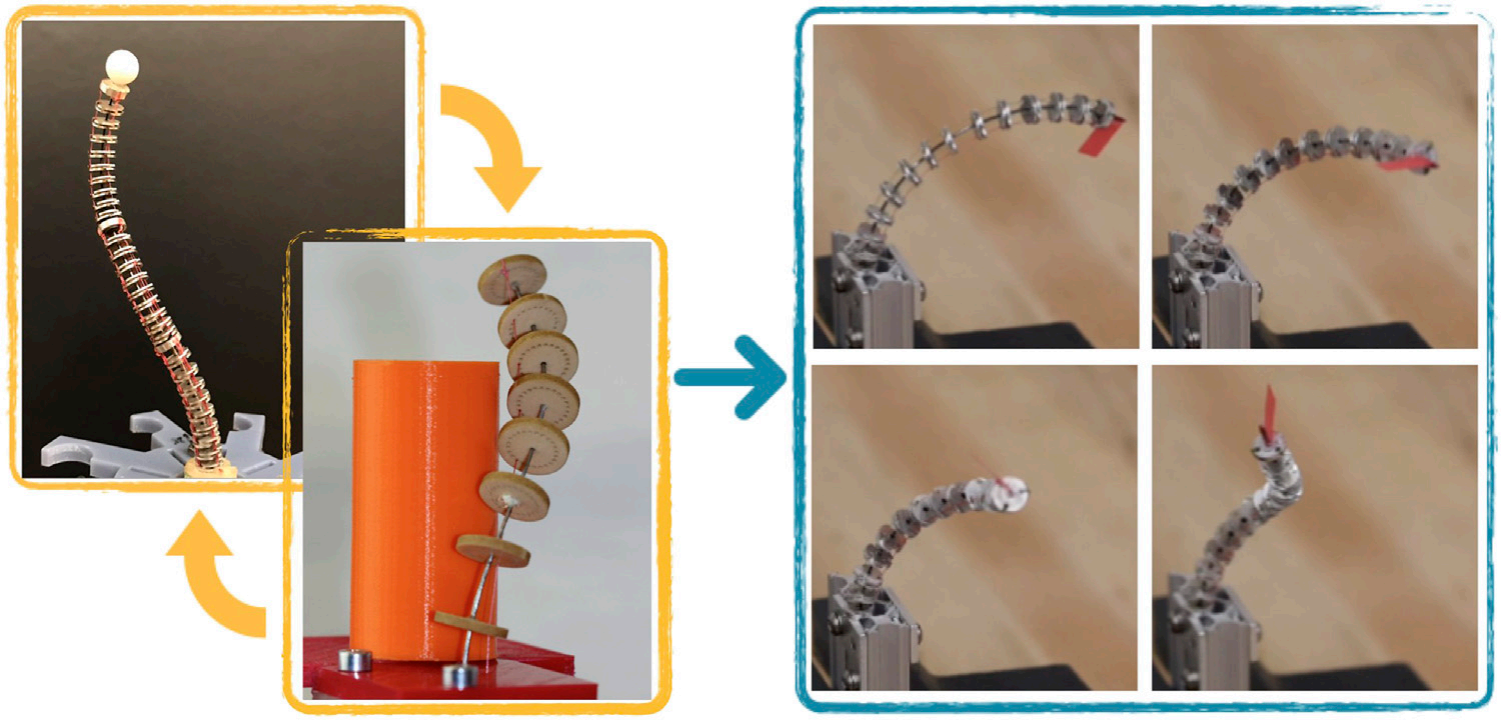
Envisioned tendon-driven continuum robot (on the right) as combination of extensible segment TDCR [top left, from (Amanov et al., 2019)] and TDCR with helical routing [bottom left, from (Starke et al., 2017)].

In medical and *in situ* inspection applications, robots are required to operate in a constrained environment and follow tortuous path. To achieve these high motion capabilities at the end effector but also of the whole robot body is desirable, in order to enable obstacle avoidance, follow-the-leader (FTL) deployment, where the robot structure moves along a predefined path, and to be able to perform a task, i.e. positioning and orienting tools at the robot tip. A promising category of robots, which are scalable to a small size, dexterous, and flexible enough to provide these capabilities comes to the fore; continuum robots. In Burgner-Kahrs et al. (2015), a continuum robot is defined as an actuatable structure, which forms curves with continuous tangent vector. Amongst the existing classes of continuum robots, tendon-driven continuum robots (TDCR), depicted in Figure 1, are one of the most frequently considered.

The capability to form a curve with continuous tangent vector is beneficial for FTL deployment. However, the motion capability of current designs of TDCR is limited, especially w.r.t. FTL behaviour. They are highly dependent on the TDCR design regardless of the modelling, sensing, and control of the FTL deployment. Therefore, a closer look at TDCR designs that improve motion capabilities is worthwhile.

### 1.1 Related Work

A TDCR is usually composed of a flexible backbone along which tendons are guided using spacer disks, these last ones being fixed to the backbone. It is actuated by changing the tendons’ length and applying tendon tensions, which cause a reversible deformation of the backbone. This actuation strategy is referred to as extrinsic, since the actuators used to deform the backbone are not embedded within the manipulator’s body. Extrinsic actuation allows for small diameter to length ratios of the robot and reduces the reflective inertia of the manipulator. TDCR are usually designed with several actuatable segments, by routing additional tendons along the backbone. These tendons are attached at specific intermediate disks fixed on the backbone, delimiting the so-called segments, and routed to the robot base where the actuators are located. The design of most of them uses two or three segments. As a result, they are good candidates for a small-scale robot with high motion capabilities, maneuverability, and interesting FTL deployment properties due to their high number of actuated dof. However, a high number of segments comes at the expense of complex tendon routing, high friction between the tendons, and a bulky actuation unit. Improving the robot motion capabilities can be performed by enhancing the capability of FTL deployment and the workspace.

Regarding FTL deployment, TDCRs have been considered to follow paths composed of sections with planar constant curvature (Palmer et al., 2014; Kang et al., 2016; Neumann and Burgner-Kahrs, 2016; Amanov et al., 2019; Gao et al., 2019). A path can be followed by deploying the TDCR along the desired path and modifying the tendon tensions, which subsequently change the curvature of the segments. To improve accuracy during deployment, a TDCR with extensible segments, which is depicted in top left image in Figure 1, is proposed by Nguyen and Burgner-Kahrs (2015) and further developed by Amanov et al. (2019).

However, due to the fact that existing TDCRs rely on straight tendon routing, only paths composed of sections with planar and constant curvature can be followed. Therefore, to facilitate general path-following, a TDCR design would require more dofs in task space (Amanov et al., 2019). As a consequence, following spatial tortuous paths requires at least two segments and for a more complex spatial path significantly more segments are required. Aside from the challenges caused by using high number of segments, e.g. complex tendon routing and increased friction, FTL deployment accuracy of standard TDCR decreases at the transition between two sections of the path as shown by Amanov et al. (2019).

As an interesting side-effect, the use of extensible segments enhances the workspace and position reduncancy of a TDCR. Existing works in the literature mainly focused on investigating the workspace improvement, i.e. which end-effector positions can be reached by the robot end-effector using the actuated dofs (Blessing and Walker, 2004; Walker et al., 2006; Amanov et al., 2019). A workspace volume increase of 22.5% compared to a TDCR with constant segment length was obtained by Amanov et al. (2019). The positive impact of the segments extensibility on the workspace is also clearly demonstrated by Li et al. (2017). Position redundancy, i.e. the capability of changing the robot configuration while staying at a desired end-effector position, was less considered but is also an important performance metric. It indicates the range of achievable robot tip orientations for the considered tip position, which is critical for manipulation (Simaan et al., 2009) and inspection (Goldman et al., 2013) tasks. The redundancy can also be leveraged for respecting anatomical constraints with the robot body (Gao et al., 2019)), or for minimizing the potential energy stored in the backbone (Moll and Kavraki, 2004). It was demonstrated by Wu et al. (2017) that the position redundancy is strongly impacted by the number of actuated dofs. Considering extensible segments improves also the position redundancy distribution at the workspace center Amanov et al. (2019).

The workspace and steerability of TDCR can also be enhanced by using non-straight tendon routing. In particular, helical tendon routing, which is depicted in the bottom left image in Figure 1, has been used to obtain TDCR configurations, which are not possible with straight tendon routing. As a result, a larger variety of tip orientations can be achieved in the workspace (Gerboni et al., 2015) or different position can be reached, while keeping the same orientation (Rucker and Webster, 2011). Further, using an additional helical tendon to existing straight tendon routing can lead to a fourfold increase of workspace volume and modification of workspace shape as demonstrated by Starke et al. (2017). However, the prototypes presented in Gerboni et al. (2015) and Starke et al. (2017) combine fixed helical and straight tendon routing and may lead to complex tendon routing, especially if several segments are stacked. A similar approach is the configurable tendon routing in (Barreiros et al., 2019), where non-straight tendon routing can be realized. However, the chosen fixed tendon routing is a design parameter and cannot be controlled during the operation of the TDCR.

To conclude, the current design paradigm, i.e. stacking segments with fixed tendon routing, has reached its limit for improving motion capabilities while achieving small scale manipulators. Although different non-straight tendon routings are considered to achieve larger workspaces and, therefore, higher motion capabilities, current tendon routings are fixed and only used as design parameters. We propose to exploit the tendon routing as an additional dof in order to induce torsion in the backbone, thus exploiting all significant deformation modes of a TDCR segment. It is our hypothesis that taking advantage of the fourth dof has a significant impact on the motion capabilities and, therefore, on the FTL deployment as well as position and orientation capabilities of a TDCR.

### 1.2 Contribution

In this paper, we propose a design of TDCR segment which leverages advantages of both extensibility and variable helical routing, cf. Figure 1. We present our progress in the development of a TDCR with four dof per segment. We introduce the design and evaluate its merits by several assessments in simulation and with real prototypes. In particular, the contributions of this paper include:

• A mechanism able to continuously change the tendon routing from straight to helical routing and capable of FTL deployment along a spatial tortuous path using a simple deployment strategy.
• An analysis of position redundancy and FTL behaviour using a static model considering four dof.
• As minor contributions, an area-based error measure for FTL deployment and a different view on spacer disks to exploit the provided passive dof.

### 1.3 Organization

The paper is structured as follows. We first introduce the conceptual design of TDCR in Section 2. We then propose a static model of the robot in Section 3. The FTL deployment capabilities are studied in Section 4, where the deployment strategy and the area-based error are described. The position and orientation capabilities are studied in Section 5. The results are discussed in Section 6 before concluding.

## 2 Conceptual Design

In this section, we discuss the concept of the proposed mechanical design. First, we consider spacer disks with passive dof. Second, we propose a segment design for TDCR which is capable of generating non-straight tendon routing. Afterwards, both physical prototypes are introduced.

### 2.1 FSD—Floating Spacer Disk

We introduce a categorization of spacer disks into four types. The categories are based on the most general spacer disk, referred to as FSD (floating spacer disk), which can translate along and rotate about the TDCR’s backbone.

Type-0: A spacer disk which can neither rotate nor translate along the backbone.
Type-I: A translational FSD which can only translate along the backbone but not rotate.
Type-II: A rotational FSD which can rotate while translation is prohibited.
Type-III: The last type of spacer disk can translate and rotate freely.

The first type of spacer disk, i.e., type-0, is fixed to the backbone and is a widely used spacer disk for TDCRs (Rucker and Webster, 2011; Mishra et al., 2017; Starke et al., 2017; Li et al., 2020). End disks and disks attached to the base fall into the category of type-0 FSD. Type I FSD can be found in the literature (Blessing and Walker, 2004; Kang et al., 2016; Amanov et al., 2017; Lastinger et al., 2019; Visentin et al., 2019), where the rotational passive dof is constrained due to its design. The position of each disk can be depended on the repulsion forces between them in order to distribute the disks along the backbone. Adding an additional rotational passive dof leads to an FSD with two dof, i.e., type-III FSD. An example is given by Nguyen and Burgner-Kahrs (2015). However, the design by Nguyen and Burgner-Kahrs (2015) does not utilize the rotational passive dof. Another FSD with rotational passive dof is given by type-II FSD. Since type-II FSD and type-III FSD are coupled with tendons, the resulting orientation of each of them is influenced by the adjacent FSD of any type. To conclude, type-II FSD have not been realized thus far and type-III FSD have not been fully exploited.

### 2.2 FAS—Fully Actuated Segment

**FIGURE 2:**
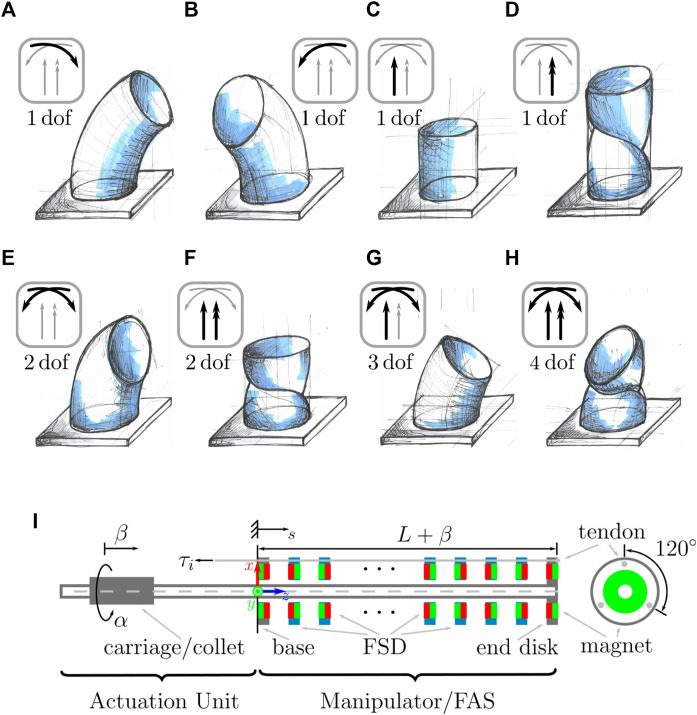
Degree of freedom of a single segment of a continuum robot represented as a blue continuum structure. **(A)** *κ*_*x*_ is a one dof bending in a plane. **(B)** *κ*_*y*_ is a one dof bending in another plane being perpendicular to the first bending plane. **(C)** Using *β*, the change of length as extension or contraction of a segment is one dof. **(D)** one dof twist along the centerline of the segment induces by *α*. **(E)** Bending in both bending planes results in two dof. Note that bending in one plane, e.g. $\kappa =\sqrt{\kappa_{x}^{2}+\kappa_{y}^{2}}$ with an additional rotation of the banding plane, e.g. *ϕ* = arctan2 (*κ*_*y*_, *κ*_*x*_), yields the same two dof result, where *κ* and *ϕ* are directly actuated. **(F)** Variable segment length with the ability to independently twist results in two dof. **(G)** Variable segment length additional to both bending are in total three dof. **(H)** The four dof envisioned design can actuate all degree of freedom which include both bending, variable segment length, and twisting. **(I)** Schematics of a TDCR prototype composed of one FAS. The green and red colors indicate the alternating poles of the magnets used for the type-III FSD. This TDCR prototype is capable of all illustrated motions.

Here, we present the general idea of our proposition of TDCR segment with four actuated dof, i.e. bending in two directions, extension and twist, obtained using extrinsic actuation. A schematic design is depicted in Figure 2I. The proposed segment design is partially based on the extensible segment TDCR design described in our previous works by Amanov et al. (2019). It uses flexible, slender and non compressible tubes as the backbone, which shear deformations are typically negligible. Our segment design, called FAS (Fully Actuated Segment), allows then to actuate all significant deformation modes of the backbone. Furthermore, connections to concentric tube continuum robots (CTCR) are made. We kindly refer to Gilbert et al. (2016) and Mahoney et al. (2016) as well as to Grassmann et al. (2020) for more details on CTCR. For visual aid, Figure 2A to Figure 2H illustrate the effect of each dof and their combinations on a continuous slender structure.

#### 2.2.1 Bending—two dof

At least three tendons are equally distributed around the elastic backbone and attached to the end disk. All tendons are routed through each FSD. As the segment backbone cannot be compressed, applying tendon tension, denoted *τ*_*i*_ for *i*th tendon, results in bending of the segment only. This bending provides two dof, i.e. *κ*_*x*_ and *κ*_*y*_, which is the bending around the *x*-axis and *y*-axis, respectively. The bending and their combination are illustrated in Figure 2A, Figure 2B, and Figure 2E.

Note that an in-compressible segment can also be actuated by specifying tendon displacements. However, tendon displacements are coupled. For instance, a planar one dof bending requires two tendons and, in this case, therefore, two actuators. In turn, these two dof in actuation space lead to one dof in arc space and one dof manifold in the task space, i.e. the planar curve with *y* and *z* coordinates can be parameterized with one parameter, e.g. *κ*_*x*_. Further, spanning a perpendicular bending plane in the *zx*-plane, leads to another one dof manifold in the task space, which can be parametrized with *κ*_*y*_. Combining both bending planes, where four actuators are used (four dof in actuation space), leads to a two dof manifold in the task space creating a curved plane in the spatial space. Note that each point on the two dof manifold has a fixed orientation and, therefore, the orientation is uniquely determined by the arc parameters *κ*_*x*_ and *κ*_*y*_. For the sake of completeness, an alternative derivation can be given by considering one bending and one rotation of this bending plane, see caption of Figure 2E.

#### 2.2.2 Extension—one dof

The backbone can be attached to a carriage at the proximal end of the backbone. The carriage can be translated in the actuation unit similar to a CTCR, cf. Amanov et al. (2019) and Grassmann and Burgner-Kahrs (2019). Therefore, the translation *β* can be extrinsically actuated. In order to enable rearrangement of the FSD, a repulsion force between them is desired. For instance, permanent magnets (Nguyen and Burgner-Kahrs, 2015) or springs (Blessing and Walker, 2004) can be used. Note that springs might prohibit rotation around the backbone due to high torsional stiffness. Here, we consider FSD equipped with permanent magnets with alternating pole orientation. They automatically distribute along the segment due to magnetic repulsion forces as Nguyen and Burgner-Kahrs (2015) previously demonstrated. The range of extension and contraction of each segment depends on the tube length and the thickness of the FSD (Neumann and Burgner-Kahrs, 2016). Figure 2C illustrates this one dof.

#### 2.2.3 Twisting by Rotation—one dof

Rotating the backbone results in rotating the end disk, which is a function of *α* and the other states of the TDCR, since the end disk is rigidly attached to the backbone. In Figures 2A,D continuously deformed structure illustrates the one dof twisting by rotation. As the end disk is rotated and each intermediate type-III FSD has a passive rotational dof, each intermediate type-III FSD will be rotated due to their interaction with the tendons. Consequently, the tendons are not routed straight any more. Applying tendon tension *τ*_*i*_ along the non-straight tendon routing then leads to twisting as shown by Rucker and Webster (2011). To conclude, the design has the ability to generate variable non-straight tendon paths by varying *α*.

To the best of our knowledge, the rotation *α* leading to variation of relative rotation of the intermediate spacer disks has never been proposed before. The designs by Mishra et al. (2017); Visentin et al. (2019); Li et al. (2020) also introduce a rotation. However, the additional rotation can be seen as a rotation of the base or as rotation of the bending plane, see caption of Figure 2E. More importantly, these designs cannot generate non-straight tendon routing.

### 2.3 Prototypes

**FIGURE 3:**
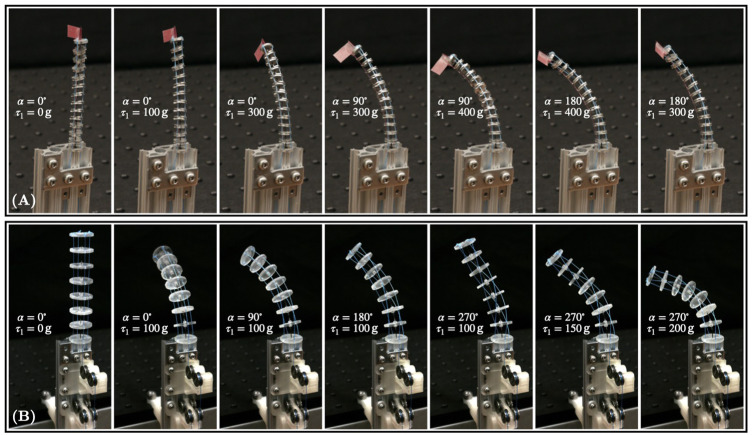
TDCR prototypes. Both prototypes are loaded with different weights on one tendon for different rotation *α*. **(A)** First prototype is composed of type-III FSD with fixed overall length of 60 mm. **(B)** Second prototype is composed of type-II FSD.

To highlight the capabilities of the proposed design, we study its merit through two different prototypes throughout this paper. The first prototype combines type-III FSDs with 4dof to obtain a FAS prototype. The second prototype with fixed segment length has several type-II FSDs. Both prototypes are depicted in Figure 3.

#### 2.3.1 FAS Prototype With Type-III FSD

As illustrated in Figure 2 and shown in Figure 3, this prototype is composed of three tendons, one backbone, and twelve FSDs. The tendons are microfilament braided threads made of Spectra Fiber^®^ (diameter of 0.28 mm) and fixed to the end disk. A superelastic Nitinol rod (metal alloy of nickel and titanium, diameter 0.635 mm,Young’s modulus 58 MPa) is used as the backbone. The translation and rotation of the backbone are manually actuated. We use a collet to maintain the rod at a desired deployed length, emulating an actuation carriage. The minimum segment length is 23 mm. The magnets composing type-III FSD are ring neodymium magnets (grade N45, outer diameter 5 mm, thickness 1 mm, inner diameter 1.5 mm) as designed in Amanov et al. (2019). All FSD have a thickness of 1.5 mm, a diameter of 7 mm, and contain three holes for the tendons to pass through, spaced with an angle of 120° at a distance 2.5 mm from the center. Both base and end disk are equipped with a magnet of the same type used for the intermediate type-III FSD. The end disk is rigidly attached to the end of the Nitinol rod. Each disk is machined and made out of aluminium.

To achieve the minimum segment length, the magnetic repulsion forces must be overcome because the distances between the type-III FSD are decreased. Note that the backbone is not compressible. However, its length can be controlled by a translational motor or a set screw. By treating the translational dof as a passive dof, we can find the minimal tendon forces to achieve the minimum segment length. The applied tendon force for each tendon is achieved by adding 120 g of weights. Actuating the segment length with a translational motor and assuming that actuation of the segment length is independent from the tendon displacement, the applied translational force of the translational motor would be 3.6 N.

#### 2.3.2 Prototype With Type II FSDs

The second prototype is composed of four tendons, one backbone, and six type-II FSDs. All tendons are fixed to the end disk which is rigidly attached to the end of the backbone. Braided threads made of Spectra Fiber^®^ (diameter of 0.28 mm) are used for the tendons. The backbone is a Nitinol rod (diameter 0.635 mm) which can be rotated by rotating the collet. All six type-II FSDs including the end disk have a thickness of 2 mm, a diameter of 20 mm, and have four holes for the tendons to pass through, spaced with an angle of 90° at a distance 7 mm from the center. The distance between two adjacent disks measured from midpoint to midpoint is 13 mm, giving a total segment length of 93 mm. Each disk weighs 0.69 g and is 3D printed with a stereolithography printer (Formlabs^®^, Clear resin). To prevent the passive translational one dof of an FSD, each type-II FSD is sandwiched between two stoppers. All stoppers are made from the same material as the spacer disk and are rigidly attached to the backbone. Figure 3 depicts the physical prototype with exemplary configuration.

For experimental assessment, a pretension of 0.01 N is applied to all tendons. Each tendon can be loaded manually with precision weights to bend the flexible backbone.

## 3 FAS Modeling

To investigate the proposed FAS design we derive a static model. The continuous backbone is represented by using a lumped parameterization approach, where it is approximated by a finite number of parameters. We adopt the nomenclature defined in Rao et al. (2021), where a segment with *n* disks is divided into a series of subsegments *i* of length *l*_*i*_ = (*L* + *β*)/*n*, where *i* = 1, 2, *…* , *n*. Each subsegment consists of disk *i* and the portion of the backbone between disks *i* and *i* − 1. The piecewise constant-curvature (PCC) approximation is applied to each subsegment, modeling the segment as a series of mutually tangent arcs. We assume that the shear, elongation, and compression of the backbone are negligible and that the disks are equally distributed in the inserted backbone length, as done in Chikhaoui et al. (2019). We consider frictional forces acting between the tendons and disks, modeled according to the Coulumb friction law. Additionally, we consider gravity acting on the robot due to the weight of the disks. We model the tendon paths as partially constrained, i.e. the portion of the tendon between two disks is represented by a straight line segment. We adapt the model proposed in Yuan et al. (2019) to account for variable tendon routing.

### 3.1 Kinematics

**FIGURE 4:**
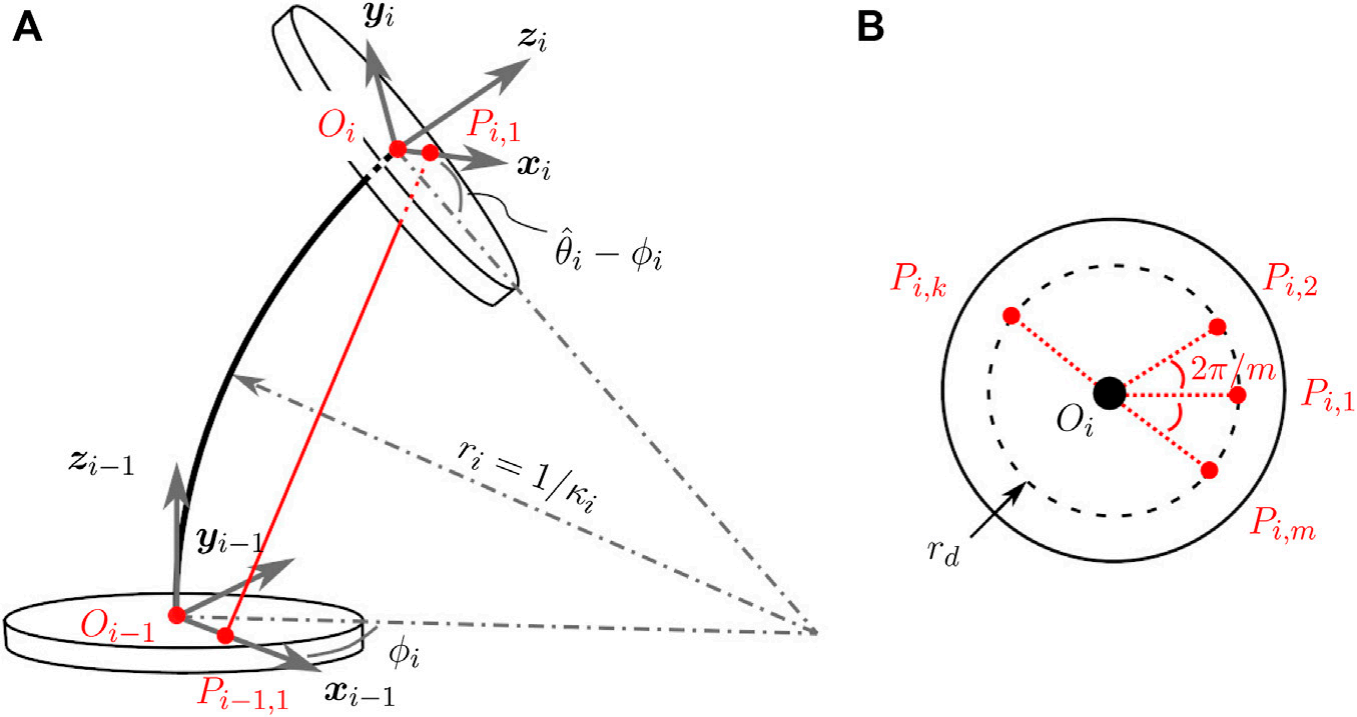
**(A)** Diagrammatic representation of two disks *i* and *i* − 1 and the portion of the backbone between them. The corresponding frames attached at *O*_*i*−1_ and *O*_*i*_ have been indicated. Only one tendon has been marked red for clarity. **(B)** The *m* tendons are arranged concentrically around the backbone at a distance of *r*_*d*_.

The center of each disk *i* is denoted by *O*_*i*_. A reference frame is attached to the base of each subsegment *i* at *O*_*i*−1_ consisting of the local ***x***_*i*−1_, ***y***_*i*−1_, ***z***_*i*−1_ axes, shown in Figure 4A. The bending curvature components along the *x*-axis and *y*-axis are represented by *κ*_*x*,*i*_ and *κ*_*y*,*i*_, while *θ*_*i*_ represents the geometric twist angle about the local *z*-axis, independent of the backbone torsion. The backbone is rotated by *α* in the actuation unit. This rotation results in each intermediate disks rotating about their local *z*-axis due to the tendon interactions. The rotation of each disk *θ*_*i*_ around the backbone is a passive dof and relative to the backbone rotation due to torsion. We assume that this rotation can be expressed as a function of the index of the disk and written as

$$\theta_{i}=\alpha f\left(i\right).$$ (1)

Since the base is fixed, *f* (0) = 0 and *f*(*n*) = 1 are assumed. Then the relative angle of rotation between the disks is given by $\left(\theta_{i}-\theta_{i-1}\right)$ . Therefore, each subsegment experiences bending and twisting, represented by the configuration parameters $X_{\mu ,i}=\left[\kappa_{x,i},\;\kappa_{y,i},\;{\hat{\theta }}_{i},\;l_{i}\right]$ , where ${\hat{\theta }}_{i}=\theta_{i}-\theta_{i-1}+\epsilon_{i}$ . The angle of twist *ϵ*_*i*_ is due to tendon tension imposed by the bending, i.e. *κ*_*x*,*i*_ and *κ*_*y*,*i*_, whereas both geometric twist angle, i.e. *θ*_*i*_ and *θ*_*i*−1_, are due to *α*. Therefore, ${\hat{\theta }}_{i}$ considers both contribution from the kinematics and statics.

Using *ϕ*_*i*_ = arctan2 (*κ*_*y*,*i*_, *κ*_*x*,*i*_) and $\kappa_{i}=\sqrt{\kappa_{x,i}^{2}+\kappa_{y,i}^{2}}$ for the sake of simplicity, the position of the end of a subsegment *i* written w.r.t. to the reference frame attached to disk *i* − 1 is given by

pii−1=cosϕiκi1−cosκilisinϕiκi1−cosκili1κisinκili. (2)

The corresponding transformation matrix from the frame at disk *i* to disk *i* − 1 is given by

Tii−1=RzϕiRyκiliRz−ϕi+θ^iPii−10⊤1, (3)

where ***R***_*y*_ and ***R***_*z*_ are two of the basic rotation matrices with index indicating the axis of rotation.

The transformation between any two reference frames is given by the product of transformation matrices between pairs of consecutive disks. The tendons are numbered in an anti-clockwise manner and are arranged uniformly at a distance of *r*_*d*_ with an angle of 2*π*/*m* between them, as shown in Figure 4B. The location of tendon *k* at disk *i* for a robot with *m* tendons is denoted by *P*_*i*,*k*_ and its coordinates w.r.t. the frame of reference at disk *i* − 1 is denoted by the ${vector}^{\;i-1}\vec{O_{i-1}P_{i,k}}$ . This vector is given by

Oi−1Pi,k⃗i−1=Tii−1rd⁡cos2πmk−1rd⁡sin2πmk−101⊤. (4)

### 3.2 Static Modeling

While the backbone representation maps the curvatures to the resulting pose in the task space, static modeling is required to obtain the curvatures for the corresponding input tensions. We do so by calculating the net moment^*i*−1^ ***M***_*i*_ acting on each subsegment *i* caused by the forces acting on a subsegment. These forces include gravity acting in the negative *z*-axis due to the weight of the disks, frictional forces written using the Coulumb friction model and the forces applied on the tendons, as described in Yuan et al. (2019). We then use the Hooke’s law to relate the net moment to the resulting curvature and torsion of each subsegment using the following equation (Rao et al., 2021),

Mii−1=RzϕiRyκiliEIxx000EIyy000GJ/li0κiϵi, (5)

where *E* and *G* are the Young’s and shear modulus, *I*_*xx*_ and *I*_*yy*_ are the second area moments and *J* is the polar second moment of cross sectional area.

### 3.3 Numerical Solution

The equilibrium Eq. 5 constitute an implicit static model of FAS as a system of 3*n* non-linear equations of the form

$$G\left(X_{\mu },pE,u\right)=0,$$ (6)

where ***X***_*μ*_ is the state space vector, such that $X_{\mu }=\left[\begin{array}{ccc} X_{\mu ,1} & \cdots & X_{\mu ,n} \end{array}\right]$ , ***p***_*E*_ is the end-disk position and ***u*** is the vector of actuation inputs. This vector contains normally all the tendon tensions *τ*_*i*_ applied at the robot base. However, we can reduce in our case the dimension of the actuation space spanned by these tensions by looking at the FAS properties. Since the backbone is assumed to be inextensible and the torsion deformations due to tendon actuation can be reasonably neglected, as demonstrated later on, the *m* tendons can only control two bending degrees of freedom independently. As a result, the tendon actuation space can be represented by two variables. We chose here to express the tendon tensions with polar coordinates. We consider that pulling on the *m* tendons is equivalent to pulling on one single tendon, located at a distance *r*_*d*_ from the backbone, an angle *γ* around the backbone tangent from tendon 1, with a force *T*:

$$\tau_{i}=T\cos\left(\frac{2\pi }{m}\left(i-1\right)-\gamma \right)+\frac{T_{t}}{m}$$ (7)

The pretension term *T*_*t*_ is a predefined value added to prevent slack in the tendons and to ensure that constraint $\sum_{i=1}^{m}\tau_{i}=T_{t}$ holds. As a result, the tendon actuation can be parametrized using *γ* and *T* only. Considering now the actuated backbone rotation and translation, the actuation space of the FAS is four dimensional and spanned by $u={\left[\begin{array}{cccc} T & \gamma & \alpha & \beta \end{array}\right]}^{⊤}$ .

The implicit static model (6) is used to solve numerically the forward and inverse static of the FAS. The forward statics are obtained by fixing ***u*** and solving the equations for ***X***_*μ*_, which allows in turn to obtain ***p***_*E*_. The inverse statics are obtained by fixing ***p***_*E*_, as well as three actuation inputs, and solve (6) for ***X***_*μ*_ and the remaining actuation input. Both forward and inverse static models are solved using a Newton-Raphson algorithm on Matlab.

### 3.4 Model Verification

In this section, we verify our model. First, the influence of the backbone rotation on the disk rotation is determined. Afterwards, robot shapes are measured for different configurations. Some of them are used to calibrate the model. Finally, the model is evaluated.

#### 3.4.1 Identifying Tendon Routing

To quantify the non-straight tendon routing obtained after rotation of the backbone, we identify experimentally the function *f*(*i*) in (1) by using the first physical prototype, see Section 2.3.1. Since the tendons are only pre-tensioned, the segment is straight. To measure the angle between the base and the different FSDs, we add a protractor scale at the base and attach an outward-pointing marker on each FSD. We read off the current angle of an FSD for a given *α*. The length of the segment is set to 65 mm, while the angle *α* is varied, i.e. *α* ∈ { − *π*/2 rad, − 4*π*/3 rad, 3*π*/2 rad}.

**FIGURE 5:**
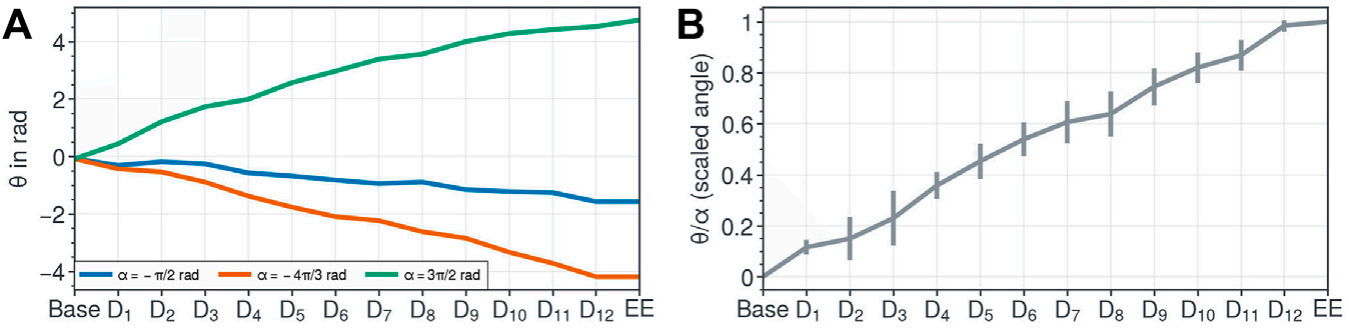
Course of disk orientation for tendon routing assessment. Orientations in rad of each disks indicate a helical tendon routing. **(A)** Three different rotations of the end disk *EE* (type-0 FSD) causing change of the orientation of each disk *D*_*i*_, where *D*_1_ is the proximal type-III FSD, *D*_1_ is the distal type-III FSD, and all other type-III FSD are in between with increasing *i*. The blue, red, and green curve corresponds to disk rotation induces by *α* = −*π*/2 rad, *α* = −4*π*/3 rad, and *α* = 3*π*/2 rad, respectively. **(B)** Normalized angles of the three different rotations indicate a linear trend.

Figure 5 shows the orientation of each FSD and the end disk w.r.t. the base. The angles of the FSD, normalized w.r.t. *α* and indexed from robot base to tip are 0 ± 0, 0.12 ± 03, 0.15 ± 08, 0.23 ± 11, 0.36 ± 05, 0.45 ± 07, 0.54 ± 07, 0.61 ± 08, 0.64 ± 09, 0.74 ± 07, 0.82 ± 06, 0.87 ± 06, 0.98 ± 02, and 1 ± 0, respectively. We deduce from the above that *f*(*i*) is a linear function, equal to *i*/*n*, when the robot is in a straight configuration. In the rest of the paper, we will assume that this function stays linear when the FAS is bent. While this is an approximation of the real distribution of the disks rotation, it leads to a static model with reasonable accuracy as demonstrated later. Hence, the type of non-straight tendon routing is a helical tendon routing for the straight configurations with pre-tension. Note that helical curves in space are characterized by a smooth curve, e.g. tendon routing, with tangent lines at a constant angle to a fixed axis, e.g. backbone. Therefore, the prototype is capable of generating variable helical tendon routing. We identify two main causes of the error indicated by the error bars in Figure 5; tilting of the type-III FSD and parallax error.

#### 3.4.2 Data Acquisition

For the model verification, we use the second physical prototype, see Section 2.3.2. This prototype cannot change its segment length. However, it should be more suitable for the verification due to the absence of repulsive forces between adjacent disks and the larger radius of each FSD compared to the first prototype. Note that the required tendon tension for a specific bending is proportional to the square of the distance between the tendon holes and the backbone as mentioned by Oliver-Butler et al. (2019). Therefore, the second prototype is more sensible to tendon tension variation, which can be seen in Figure 3 by visually comparing both prototypes under different tendon tensions.

The points are measured with a tactile measurement device (MicroScribe^®^ MX, Revware Systems Inc., NC, United States) for different *α* values and weights. Table 1 lists all 20 configurations. For each configuration, 20 points on the perimeter of each disk are recorded. To obtain the midpoint of the respective disk, we then use eigenvectors and apply Apollonius’ problem for given *M* ≥ 3 different points.

**TABLE 1:** Configurations for model verification assessment. The tendon tensions of *τ*_1_ are indicated by the mass of the precision weights. The weights are sampled from an uniform random between 0 and 250 g. Adapted from the machine learning literature (Bergstra and Bengio (2012)), we prefer sampling from a random distribution over a grid search approach

*α*			
0 rad	−*π*/2 rad	*π* rad	−3*π*/2 rad
89 g	213 g	106 g	187 g
242 g	20 g	181 g	132 g
214 g	92 g	145 g	15 g
127 g	113 g	131 g	230 g
178 g	42 g	203 g	178 g

The Apollonius’ problem is concerned with the construction of circles that are tangent to any combination of three given objects, where an object represents a point, line, or circle (Coxeter, 1968). We take advantage of the PPP-method, i.e. three points are given. The PPP-method requires three distinguishable points and provides the sought-after midpoint. Here, we present the extended method for *M* ≥ 3 different points. Note that computing the midpoint by using the mean value of *M* different points will lead to a high error with high variance. The equation of a circle for a given 2-dimensional point with index *i* is ${\left(x_{i}-x_{m}\right)}^{2}+{\left(y_{i}-y_{m}\right)}^{2}=r$ , where the midpoint has the index *m* and the unknown radius of the circle is *r*. By expanding out the equation, reordering the values, and using the substitution $h=x_{m}^{2}+y_{m}^{2}-r$ , the equation $h+x_{m}\left(-2x_{i}\right)+y_{m}\left(-2y_{i}\right)=x_{i}^{2}+y_{i}^{2}$ can be obtained. For each measured point, a linear equation is obtained leading to a system of linear equations given by

$$\left[\begin{array}{ccc} 1 & -2x_{1} & -2y_{1}\\ 1 & -2x_{2} & -2y_{2}\\ \vdots & \vdots & \vdots \\ 1 & -2x_{M-1} & -2y_{M-1}\\ 1 & -2x_{M} & -2y_{M} \end{array}\right]\left[\begin{array}{c} x_{m}\\ y_{m}\\ h \end{array}\right]=\left[\begin{array}{c} x_{1}^{2}+y_{1}^{2}\\ x_{2}^{2}+y_{2}^{2}\\ \vdots \\ x_{M-1}^{2}+y_{M-1}^{2}\\ x_{M}^{2}+y_{M}^{2} \end{array}\right],$$ (8)

which can be solved via pseudo-inverse. Note that *h* is an auxiliary variable and *r* can be used for a sanity check, i.e. *r* should match the radius of the measured disk plus the radius of the probe of the tactile measurement device.

To extend the above method to 3-dimensional points, we project the measured points onto a suitable plane and utilized the above method exploiting Apollonius’ problem. The transformation to the plane can be found by finding a frame, where two axes span the plane and the third axis is orthogonal to the plane. The frame can be found by computing the eigenvectors or principle components of the set of measured points. Methods like Singular Value Decomposition (SVD) or Principal Component Analysis (PCA) can be used to determine the axes of the frame. For the projection, the *z* component of each transformed points is set to zero. The found frame is used to transform the measured points before the projection and transform the midpoint back to the original coordinate system. Note that the frame of the original coordinate system can be any orthonormal frame.

#### 3.4.3 Calibration

Due to errors in assembling and manufacturing, the measured base coordinate frame does not match the robot’s base coordinate frame. We model these errors by adding rotations of *γ*_*y*_ and *γ*_*z*_ about the *y*-axis and *z*-axis. To estimate the two angles in addition to the coefficient of friction, a nonlinear unconstrained optimization problem was implemented to minimize the average of the tip errors over twelve readings. The Nelder-Mead simplex algorithm implemented in Matlab’s fminsearch function was used to perform the optimization. The values of *γ*_*y*_ and *γ*_*z*_ were obtained to be −0.0873 rad, −0.0524 rad. The friction coefficient was obtained to be *μ* = 0.30. The Youngs modulus was estimated as 58 GPa.

#### 3.4.4 Evaluation of Model Performance

We use two metrics to evaluate the performance of the model. The tip error measures the Euclidean distance of the measured position of the last disk from the position predicted by the model. The backbone shape error is the average of the Euclidean distance between the measured and predicted position of each disk. We generate a set of robot configurations by considering four different values of *α* and by actuating one tendon with different weights, see Table 1.

**FIGURE 6:**
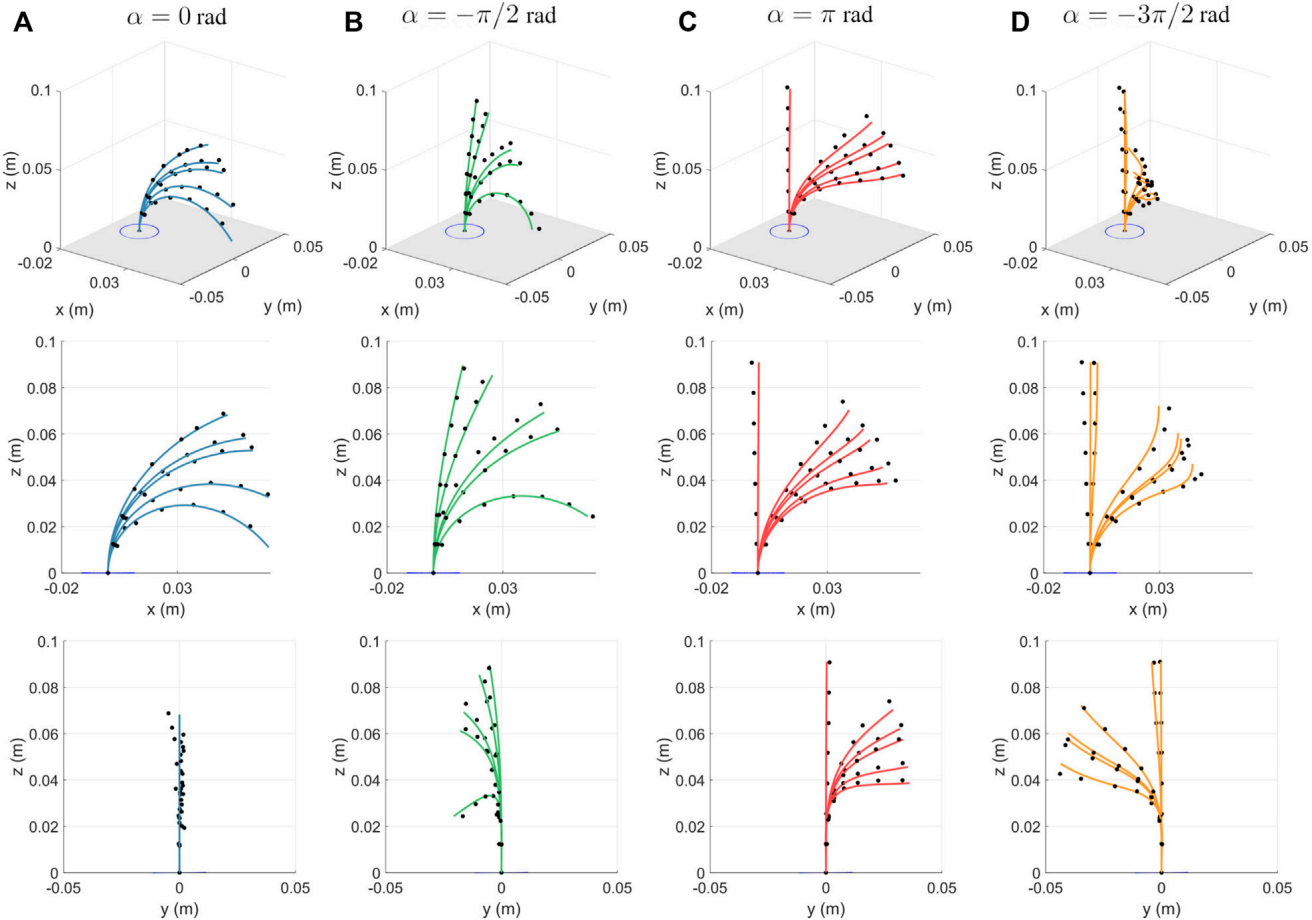
For different tension values, experimental measurements (in black dots) plotted against the backbone curve predicted by the proposed model for **(A)** *α* = 0, **(B)** *α* = − *π*/2 rad, **(C)** *α* = *π* rad, and **(D)** *α* = −3*π*/2 rad. The figures show the 3D plot (top row), projection on the *xz*-plane (middle) and the projection on the *yz*-plane (bottom).

The obtained errors for four different values of *α*, averaged over multiple readings with individual tendon tensions are summarized in Table 2. The corresponding shapes of the robot, obtained experimentally and through the model have been depicted in Figure 6. The average tip and backbone error over the 20 observations are 4 and 2.17 mm (respectively 4.39 % and 2.39% of the robot length), which are reasonable values for a first model and proof of concept of FAS.

**TABLE 2:** Average tip errors and backbone shape errors for different values of *α*. Mean and standard deviation of the errors are listed. We report the absolute errors in millimetre and relative errors in % w.r.t. segment length *L* = 93 mm.

*α* in rad	Tip error		Shape error	
	mm	% w.r.t. Length	mm	% w.r.t. Length
0	2.44 ± 1.71	2.68 ± 1.88	1.96 ± 0.56	2.15 ± 0.62
− *π*/2	3.89 ± 1.74	4.28 ± 1.91	2.14 ± 0.15	2.36 ± 0.17
*π*	3.58 ± 1.74	3.93 ± 1.91	1.91 ± 0.42	2.10 ± 0.47
−3*π*/2	6.09 ± 2.25	6.70 ± 2.47	2.68 ± 0.76	2.95 ± 0.84
$\left\{0,-\pi /2,\pi ,-3\pi /2\right\}$	4.00 ± 2.19	4.39 ± 2.41	2.17 ± 0.58	2.39 ± 0.63

The errors are the lowest for *α* = 0 rad as it only experiences in-plane bending. The largest errors occur for *α* = −3*π*/2 rad, where the out-of-plane deformations are larger. The errors could be attributed to the simplified assumptions made by the model. The model considers the tendon location at each disk to be a fixed point. However, due to manufacturing constraints the holes made for the tendons have a certain tolerance and this results in the tendon locations at each disk to vary in different configurations. Other possible sources of error could arise from inaccuracies in measurement, assembling, and manufacturing of the prototype. Along with the error between measured and predicted robot shapes, we observe that the robot backbone is not significantly twisted by the tendon tension. The maximum value of *ϵ*_*i*_, in the robot configuration involving the largest tendon forces and backbone rotation, equals 3.7*%* of *α*. This is due to the fact that the Nitinol rod composing the robot backbone is torsionally stiff.

## 4 Follow-the-Leader Deployment

In the following, we highlight the FTL deployment capabilities of the proposed design. First, we motivate a simple deployment strategy. Afterwards, this strategy is evaluated on a physical FAS prototype. Second, we further investigate this strategy in simulation with our proposed model. It has the advantage of neglecting hard-to-model phenomena, e.g. magnetic repulsion forces and slip-stick effect between FSD and backbone. Note that the repulsion forces are needed to distribute the FSD along the backbone. Therefore, we neglect the accurate modeling of the repulsion forces and assume a static equidistant distribution of FSD along the backbone to mitigate the influence of model inaccuracy of those hard-to-model phenomena.

### 4.1 Deployment Strategy

Achieving FTL behaviour requires the continuum robot to keep a constant curvature at any arc-length along the path during the deployment (Garriga-Casanovas and Rodriguez y Baena, 2018a). This has been achieved so far for multi-segment TDCR by using specific optimization routines. The displacement to be applied on each tendon is calculated at each step of the deployment in order to minimize the error between the position of the robot backbone and the path to be followed (Palmer et al., 2014). As a result, approximate FTL behaviour is obtained, which accuracy depends on the accuracy of the model used to predict the robot shape and the range of extensibility achievable by each segment (Amanov et al., 2019). Although this optimization based approach can be generally applied to any continuum robot, it is complex and can be computationally heavy. We propose here a simpler deployment strategy for TDCR and, in particular, for the FAS, that takes advantage specifically of tendon actuation.

When a single segment TDCR composed of straight-routed and fully constrained tendons is considered, and the effects of gravity and friction are assumed to be negligible, it has been shown that the tension applied by the tendon can be represented by a constant moment applied (Camarillo et al., 2008). The constant moment results in constant curvature of the backbone. As a result, by applying a constant tendon force and by progressively increasing the length of this segment, the deployed robot bends in a plane with a constant curvature. It has the same curvature at any arc-length along the path. Consequently, a one segment TDCR for which the tendon force is controlled can be used to perform perfect FTL deployments along planar paths. Similarly for the FAS, we suspect that applying constant tendon tensions during the deployment will lead to a segment with constant bending and torsional curvature. Deploying the backbone, i.e. changing *β*, while keeping the backbone rotation, i.e. *α* constant, should thus lead to FTL deployments along spatial helical paths.

### 4.2 Area-Based FTL Error

To assess the FTL deployment, we propose a measure which takes the covered projected area of the volume of the robot’s shape along a path during a deployment sequence into account. First, we define the overall covered area A given by

$$A=\overset{n}{\underset{i=1}{⋃}}Ai,$$ (9)

where $A_{i}$ and *n* are the area covered by the projected robot’s shape at the *i*th step and the number of steps to reach the end of the path, respectively. Hence, A is the projected area of the volume covered during the FTL deployment. Note that $A_{i}$ and A are defined as sets of points which constitute the respective area.

Second, we can define the area-based FTL error in terms of sets as

$$L_{\text{FTL}}=\frac{\left|\left(A_{n}\backslash A\right)⋃\left(A\backslash A_{n}\right)\right|}{\left|A_{n}\right|},$$ (10)

where $\left|\cdot \right|$ is an operator acting on the set, which gives the area a numerical value depending on the sensors used to sense the shape. The numerator is the symmetric difference, also known as the disjunctive union, which represent the difference between the ideal area $A_{n}$ and actual covered area A . The denominator normalizes the difference w.r.t. $A_{n}$ . Note that (10) represents a simplification of a volumetric error measure, which considers spatial points and a continuous time during deployment. Further note that it is assumed that the robot is extending. To consider the contraction of the robot, $A_{n}$ is replaced by $A_{1}$ , whereas A reminds the same as changing the order of the operands in (9) does not change the result.

Finally, we define the area-based FTL error $L_{\text{FTL}}$ quantified by

$$L_{\text{FTL}}=\frac{\left|A\right|}{\left|A_{n}\right|}-1,$$ (11)

which is equivalent to (10) since the nominator of (10) in combination with the overall area (9) leads to $\left|\left(A_{n}\backslash A\right)⋃\left(A\backslash A_{n}\right)\right|=\left|A\right|-\left|A_{n}\right|$ . However, (11) gives a more practical implementation.

In this paper, we use a camera to sense the shape and images as sensor outputs. To give a numerical value to the area, we construct a binary image, where all pixels occupied by the robot are white and the rest are black. The operator $\left|\cdot \right|$ adds up then the white pixels in the binary image.

### 4.3 Preliminary Study on FTL Deployment

**FIGURE 7:**
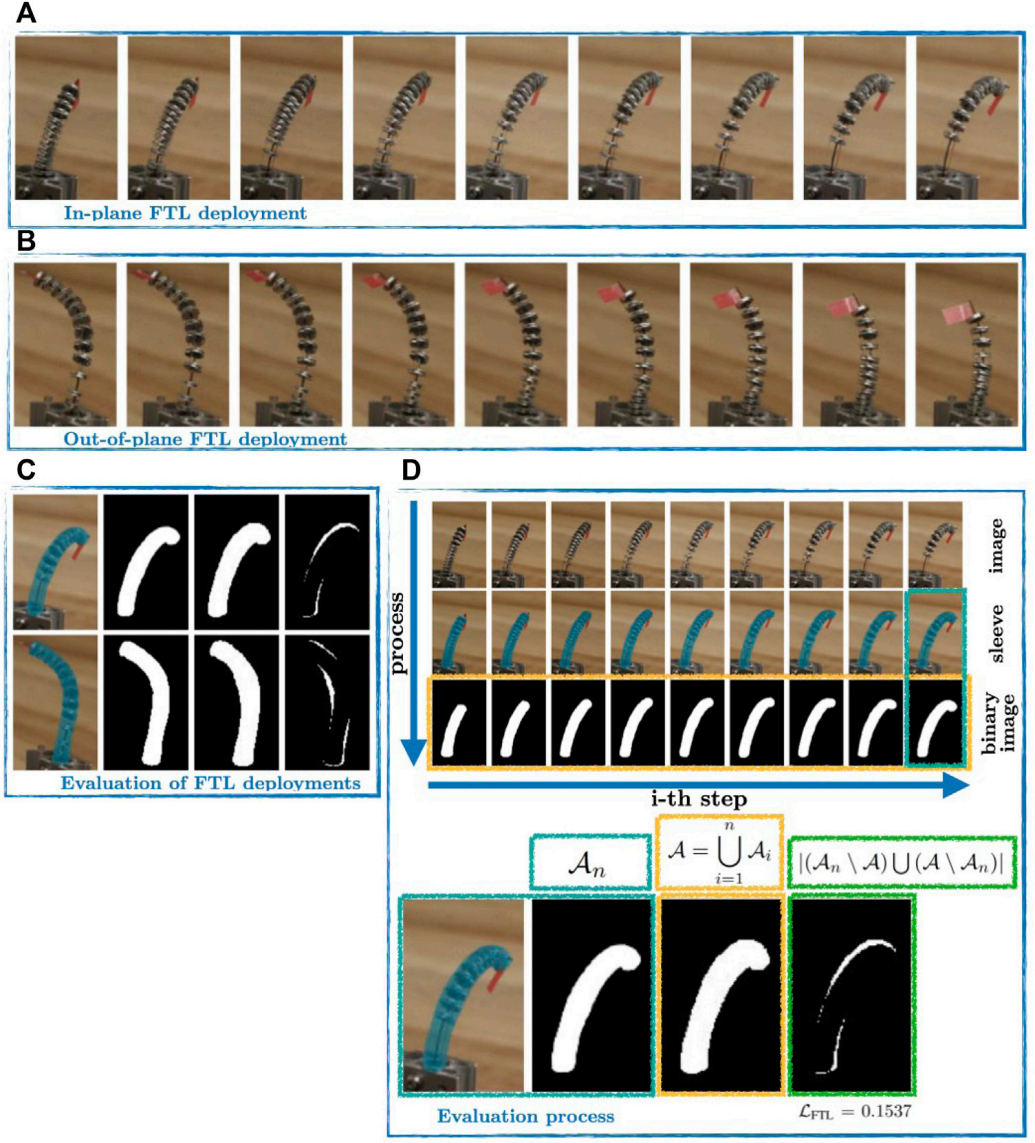
FTL deployment with physical prototype. **(A)** Sequence of an in-plane FTL deployment with straight routed tendons. **(B)** Sequence of an out-of-plane FTL deployment non-straight routed tendons. The red flag on top of the end disk is used to indicate the orientation. **(C)** Segmented binary image of the representative sequence. The reference step is contrasted with the overlapped segmented image of all images within the sequence to obtain the binary image, which will be used for $L_{\text{FTL}}$ . **(D)** The Evaluation process as described in Section 4.2 is exemplary shown for the in-plane FTL deployment. The accompanying video provides additional visual aid.

Using the FAS prototype described in Section 2.3.1, we load one tendon with an additional 5 N to the pretension. We indicate the termination point of the tendon as well as the end disk orientation using a red flag. Figures 7A,B shows a sequence of an in-plane deployment and an out-of-plane deployment, where *α* = 0 rad and *α* = −*π* rad, respectively. As can be seen from Figure 7, the orientation around the tangent vector of the backbone is constant during deployment, whereas the orientation changes continuously along the spatial path. The change in orientation indicates torsion along the backbone during the deployment.

For the evaluation of the FTL deployment, we utilize (11) and consider nine steps of each sequence, see Figure 7D. In order to consider all continuous intermediate steps between two discrete steps, we manually segment the prototype with a conceived sleeve including all disks, tendons, and the backbone. An example of a conceived sleeve is represented with a blue hull in Figure 7D. The sleeves of each sequence are overlaid and binary images are generated. The binary image indicate the occupied area representing the occupied volume during the deployment. Figure 7C illustrates the area indicated by white pixel in the binary image. We achieve an error of $L_{\text{FTL}}=0.1537$ and $L_{\text{FTL}}=0.1399$ for the in-plane and out-of-plane deployment, respectively.

Using the photographs taken from a motion sequence in (Amanov et al., 2016), the extensible segment TDCR achieves $L_{\text{FTL}}=0.2280$ , whereas a CTCR used in Girerd et al. (2020) achieves $L_{\text{FTL}}=0.3285$ in simulation and $L_{\text{FTL}}=3.2728$ with real hardware. Note that in (Amanov et al., 2016) the extensible segment TDCR has three segments and that a CTCR is typically incapable of pure FTL deployment as stated in (Gilbert and Webster, 2013). However, results give an intuition about which value of the error measure $L_{\text{FTL}}$ corresponds to an acceptable FTL deployment since it has been shown in (Neumann and Burgner-Kahrs, 2016) that an extensible segment TDCR (Nguyen and Burgner-Kahrs, 2015) is capable of FTL deployment. The achieved results $L_{\text{FTL}}=0.1537$ for the in-plane FTL deployment show a smaller error as the physical prototype has one segment. For the case *α* = 0°, the built prototype is essentially an extensible segment TDCR with one segment. Using three segments instead of one would probably lead to similar errors as the one reported by Amanov et al. (2016). Consequently, the value of $L_{\text{FTL}}$ seems reasonable. More important, the error for the out-of-plane FTL deployment being $L_{\text{FTL}}=0.1399$ is in the same range. This demonstrates that one FAS is capable of FTL deployment along spatial paths, which cannot be achieved with previously proposed TDCR segment designs.

### 4.4 Analysis of FTL Paths in Simulation

The errors of FTL behaviour observed during the FAS deployment may be due to two reasons. First, as the torsion of the path to follow is constant, the geometric twist angle *θ* must vary linearly with the arc-length along the path. As for now, the geometric twist angle *θ* is determined by the backbone rotation *α*, which is kept constant during the deployment. Second, the curvature of the segment may be constant for one deployment step but might change slightly with the segment length as the tendons are not fully constrained. They are here locally constrained by the FSD. The goals of the analysis in simulation are two folds: to study the effect of these two sources of errors and to demonstrate the range of reference path that can be followed with the FAS.

In order to do so, we first update the FTL deployment strategy. In addition to varying the inserted length of the backbone and keeping the applied tensions constant, we use variations in *α* to follow the natural change in twist of the reference path. We consider the reference path as the shape of a segment with length *l* ^∗^ actuated by pulling on the first tendon, with the central backbone rotated by *α*∗. The FTL deployment is then simulated by solving the forward static model while varying the insertion length *l*, where *l* < *l* ^∗^. The applied rotation *α* for any intermediate configuration is scaled linearly w.r.t. the inserted length and is calculated as:

$$\alpha =\alpha^{*}\frac{l}{l^{*}}$$ (12)

**FIGURE 8:**
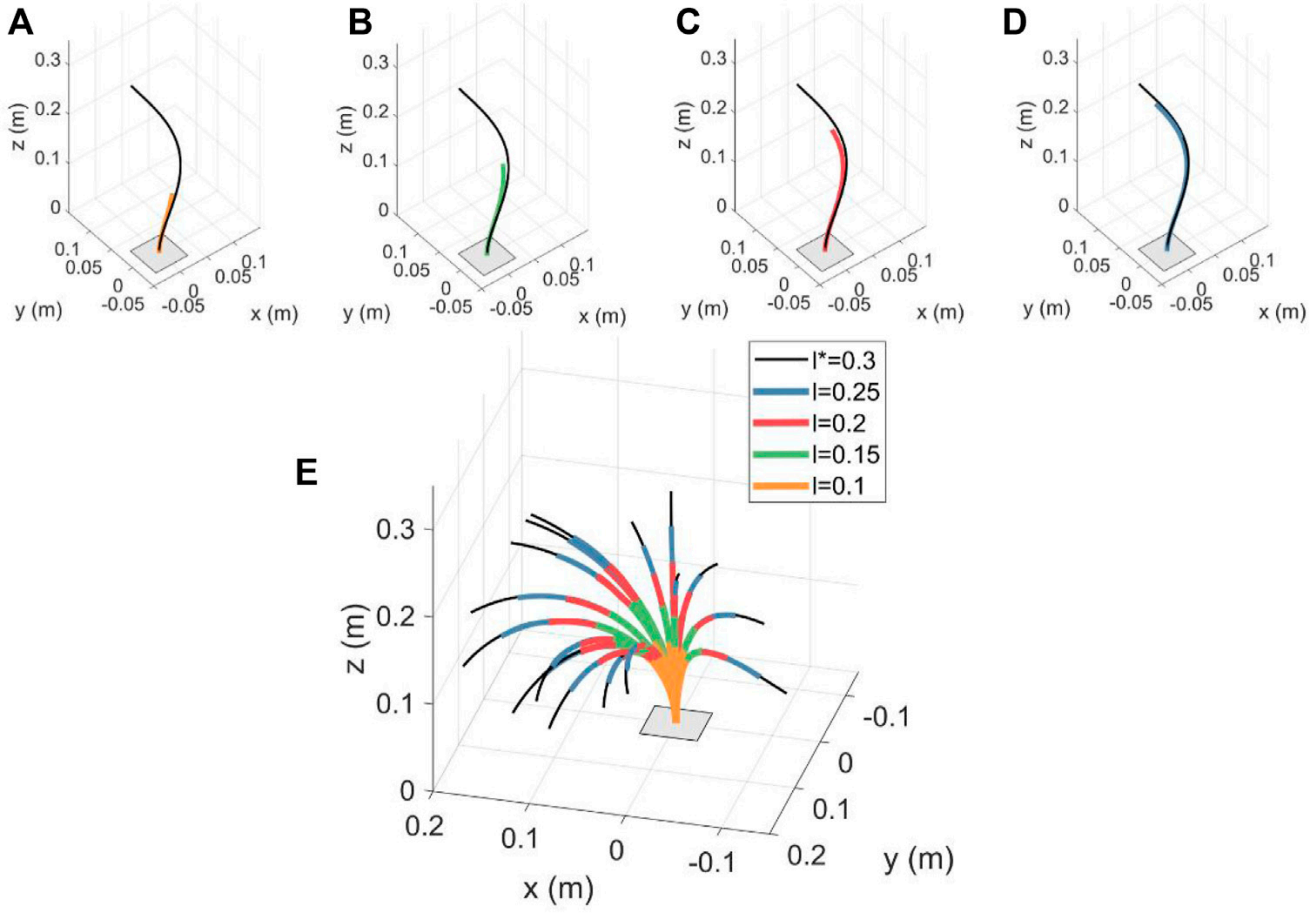
FTL deployment results for varying segment lengths and configurations. A Sequence-wise FTL deployment is shown in **(A–D)**. For α* = *π* rad and l* = 0.3 m, the desired path is shown in black. The corresponding backbone shape obtained for intermediate lengths with *n* = 10 is shown in alternate colors for **(A)** *l* = 0.1 m **(B)** *l* = 0.15 m **(C)** *l* = 0.2 m, and **(D)** *l* = 0.25 m. **(E)** FTL deployment results for 20 random values of α* and tensions. The corresponding spatial paths for *n* = 50 and the intermediate FTL deployment stages have been shown for different values of inserted length, i.e. *l* ∈ {0.1, 0.15, 0.2, 0.25 m}.

We then simulate FTL deployments with different number of disks along the backbone, to observe the effect of the tendon constraint, and for different reference paths. The obtained intermediate configurations in the FTL deployment have been shown in Figures 8A–D. We observe that for small numbers of disks, the obtained configurations do not perfectly align with the reference path. This deviation is due to the assumption of partially constrained tendon. As the tendons are not always normal to the end disk, the moments resulting from their forces, and as a consequence the backbone curvature and torsion, change with the deployed length. When the number of disks is increased such that the tendon path resembles a fully constrained design [as defined in (Rao et al., 2021)], exact spatial FTL is achieved.

The intermediate configurations have been super-imposed and indicated for different values of inserted lengths. Applying (11) to different projections of the covered area for nine equal-distance steps for a diameter of 5 mm, we get $L_{\text{FTL}}\left(xy\right)=0.257$ , $L_{\text{FTL}}\left(xz\right)=0.012$ , and $L_{\text{FTL}}\left(yz\right)=0.091$ for the projection onto the *xy*-plane, *xz*-plane, and *yz*-plane, respectively. Those values correspond to a model with *n* = 10 disks, where as $L_{\text{FTL}}\left(xy\right)=0.0581$ , $L_{\text{FTL}}\left(xz\right)=0.0259$ , and $L_{\text{FTL}}\left(yz\right)=0.0303$ correspond to *n* = 50 case. For comparison, for *n* = 50 and with the previous FTL strategy, where *α* = const. w.r.t. *l* during the deployment, we obtain $L_{\text{FTL}}\left(xy\right)=0.474$ , $L_{\text{FTL}}\left(xz\right)=0.8128$ , and $L_{\text{FTL}}\left(yz\right)=1.538$ . It is apparent that previous FTL strategy leads to higher FTL error and, therefore, varying the value of *α* linearly with length leads to better FTL performance. Further, the FTL error decreases with larger values of *n*. This reinforces the previous observation and lets us conclude that the higher the number of FSD, the better the accuracy for the FTL deployment. Finally, we can make an evident observation that the values of $L_{\text{FTL}}$ are view dependent due to the projection of the 3D volume of the robot’s shape onto a plane.

In Figure 8E, we show 20 spatial paths with α* ∈ [ − *π* rad, *π* rad] and randomized values of tensions applied to the first tendon for *n* = 50. We see that the robot observes FTL deployment for intermediate lengths along the various 3D spatial paths.

## 5 Position Redundancy

Investigating the position redundancy of the FAS involves to find the different robot configurations leading to a desired tip position, and to analyse the resulting robot shapes and tip orientations. Therefore, we first state a computational method to investigate this position redundancy. Second, the phenomena is qualitatively verified on a physical FAS prototype. Afterwards, further investigating on the redundancy are provided in simulation.

### 5.1 Numerical Analysis Method

In order to obtain the robot configurations, standard approaches rely on a heavy discretization of the actuation space (Wu et al., 2017), spanned by ***u*** in our case, or of the task space (Li et al., 2017). In the first case, the static model is solved for each set of actuation inputs, leading to a dense set of tip positions. The robot configurations leading to tip positions in the vicinity of a desired position are gathered, leading to a set of tip orientations. In the second case, the tip orientation is parametrized by rotations around specific axes and discretized for each possible tip position in the workspace. The inverse static model is then solved for each tip pose, and converges eventually toward a robot configuration in case the pose is achievable. These two methods are both computationally expensive, and their result depends on the discretization strategy and density. The task space discretization approach requires to know the rotation axis of the robot tip, which cannot be completely controled with the four dof of the FAS. Moreover, the convergence of the inverse static model depends on the initial guess provided to the numerical solver.

To alleviate these problems, we compute the robot configurations by varying one actuation input and solving the inverse static model for a desired position ***p***_*E*_ using a continuation method. The idea is to dedicate three actuation variables to maintaining the segment tip position at ***p***_*E*_, and to vary the fourth input to change the robot configuration and obtain different tip orientations. In particular, we vary the backbone rotation *α* as it has a major impact on the robot shape. The continuation method allows to vary *α* and to compute the corresponding set of robot configurations without relying on an a priori discretization and being sensitive to bad initial guesses. The set of robot configurations is considered as the smooth function B , called branch of solution of the inverse static model and defined as:

$$\begin{array}{ll} B:R & \to R^{4n+3}\\ \alpha & \mapsto {\left[\begin{array}{cccc} X_{\mu }^{⊤} & T & \gamma & \beta \end{array}\right]}^{⊤}\;\text{such that}\;G\left(X_{\mu },p_{E},u\right)=0 \end{array}$$ (13)

It is computed using a prediction and correction process. Starting from a point of the branch, the next point is predicted by incrementing the actuation input by a given step size and by taking the corresponding point along the branch local tangent ∂B/∂α . The inverse static model is then solved, starting from the predicted configuration, using a Newton-Raphson algorithm. The step size is automatically reduced in case the numerical solver has more difficulties to converge, i.e. the number of iterations required to converge increases. The process is repeated until a specified number of points along the branch have been computed. Once the robot configurations have been found, the resulting tip orientations are computed as rotation matrices ***R***_*E*_ using Eq. 3. The prediction-correction method is implemented in the Matlab toolbox called MatCont (Dhooge et al. (2008)).

Finally, analysing the tip orientations requires to have an intuitive representation of them. Service spheres provide such representation as demonstrated in Li et al. (2017). A service sphere of a given radius *r*_*S*_ is defined, where its center is located at the robot tip position ***p***_*E*_. The intersection of the robot tip tangent with the service sphere creates then a point ***p***_*S*_, which represents the tip orientation. With forward and inverse kinematics based sampling method, the service sphere is usually discretized in a finite number of patches. The patches that contain a tip orientation point are then colored, which allows an easy visualization of the possible tip orientations and the evaluation of orientability metrics. We use the service sphere representation in this work to visualize and analyse the tip orientation of the FAS. The set of tip orientations obtained after varying *α* results in a set of consecutive points ***p***_*S*_ on the service sphere, forming a one dimensional curve defined by:

$$\begin{array}{ll} p_{S}:R & \to R^{3}\\ \alpha & \mapsto p_{E}+r_{S}R_{E}e_{z} \end{array}$$ (14)

where *r*_*S*_ is the radius of the service sphere.

To illustrate the position redundancy of a segment with variable helical tendon routing, we conduct a reachability assessment. As before, we conduct a preliminary study with the physical FAS prototype described in Section 2.3.1. A more in-depth study in simulation follows afterwards.

### 5.2 Preliminary Study on Position Redundancy

**FIGURE 9:**
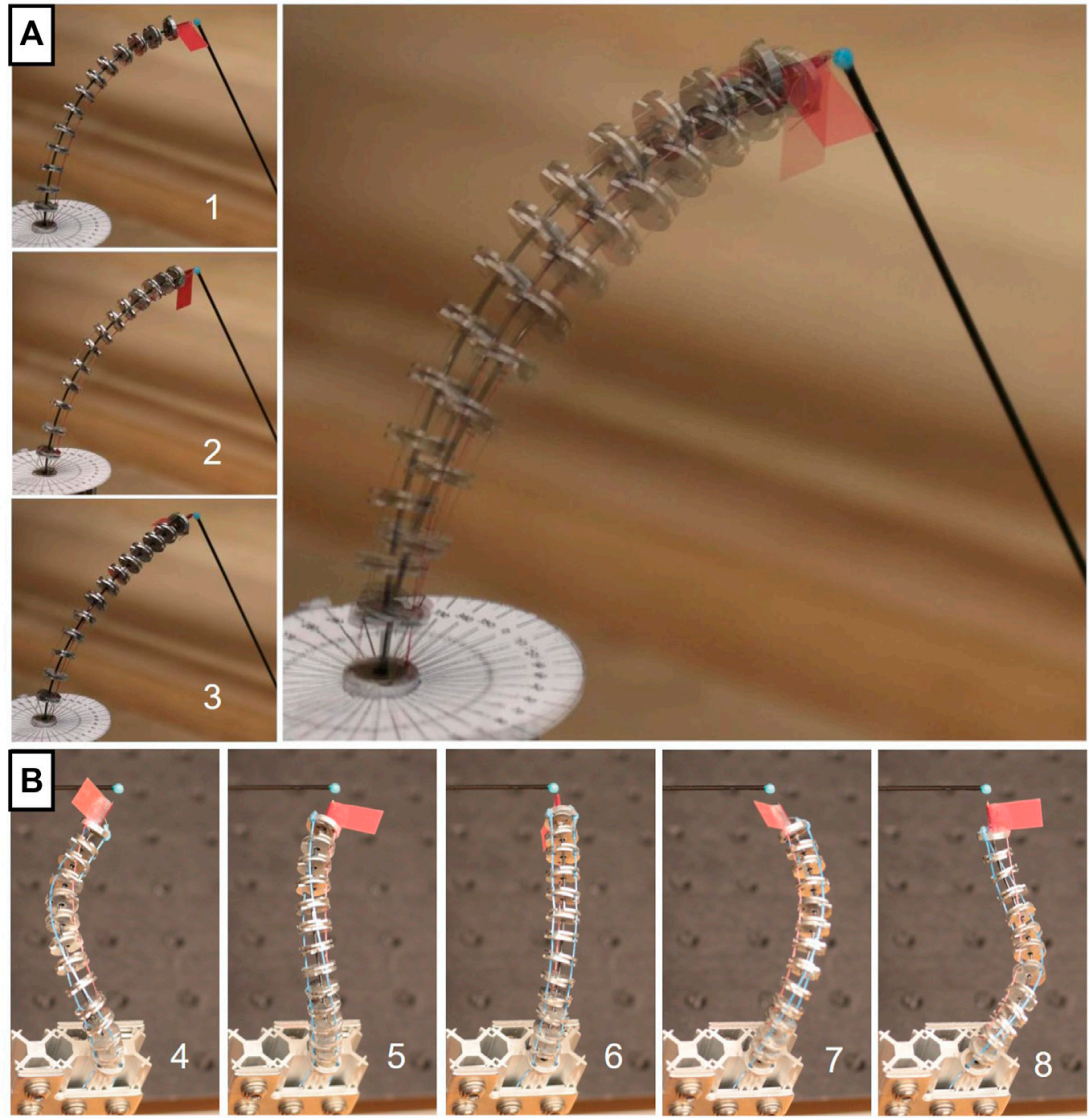
Motion capabilities indicated by position redundancy. The red flag with flagpoles of 7 mm indicates, on the one hand, the orientation of the end disk around the tangent vector of the backbone and, on the other hand, the rotation *α* of the backbone. **(A)** (Left column) Configurations with significant backbone rotation and negligible translation, see Table 3. (Right) Overlay of all configurations. The accompanying video provides additional visual aid. **(B)** More extreme configurations, requiring backbone translation.

We choose two target points in the robot workspace. Their position is indicated by a blue bead (diameter 2 mm). They lie about 58 and 51 mm in front of the prototype, and about 51 and 37 mm above the base of the prototype, respectively. Positions of the backbone origin, the hole for the first tendon in the base, and the projection of the point (blue bead) on the same *xy*-plane lies on a line, i.e. *x*-axis. The tendon force, the backbone rotation, and the backbone translation are then actuated manually in order to reach the target with different configurations. Table 3 summarizes all the configurations, configurations 1, two and three corresponding to the first target points, and the others to the second. Their realization using the prototype with one FAS is represented in Figure 9. Note that the tip position is the end disk position plus an offset, i.e. flagpoles with length 7 mm.

**TABLE 3:** Configurations for position redundancy assessment. The tendon tensions are indicated by the mass of the precision weights.

config	*τ*_1_ in g	*τ*_2_ in g	*τ*_3_ in g	*α* in rad	*L* + *β* in mm
1st	351	1	151	−2*π*/3	76
2nd	321	1	1	0	76
3rd	351	151	1	2*π*/3	76
4th	601	601	1	−2*π*	60
5th	501	401	1	−*π*	55
6th	501	1	1	0	55
7th	501	1	401	*π*	55
8th	601	1	601	2*π*	60

For the first target point, we start from configuration two and vary the backbone rotation and the tendon tension without changing the robot length. Configuration two is a pure in-plane bending in the *xz*-plane, where the first tendon is loaded with 3.21 N and *α* = 0 rad. We obtain the robot configurations 1 and 3, which lead to the same target tip position. While the robot shapes are close to each other and the tip orientation is rotated about the vertical axis of approximately − *π*/6 rad, the tip orientation varies significantly from − 2*π*/3 rad to 2*π*/3 rad. For the second target point, we start from configuration six which is again a pure in-plane bending in the *xz*-plane. The backbone is rotated with larger values, ranging from *α* = ±2*π* rad. As a result, the backbone must be translated to reach the target point, leading to configurations four to 8. Position redundancy is achieved while obtaining robot configurations with significant variations of tip orientation.

From the preliminary study with FAS prototype, we can conclude that the additional dof of the design greatly contribute to the position redundancy of the design. The dof for the extension of the segment has a negligible contribution for small values of *α*. However, leveraging *β* leads to a higher variation of *α*, and therefore to higher variations of robot shapes and tip orientations. Further, note that neither a non-extensible segment, which can only bend, nor an extensible segment, which can bend and elongate, exhibit position redundancy. In addition, note that the workspace of a non-extensible segment is a curved plane in the task space. Hence, a point on this curved plane can be reached with the FAS with different tendon tensions *τ*_*i*_ and a high variety of *α*, while *β* has a minor contribution.

### 5.3 Analysis of Position Redundancy in Simulation

**FIGURE 10:**
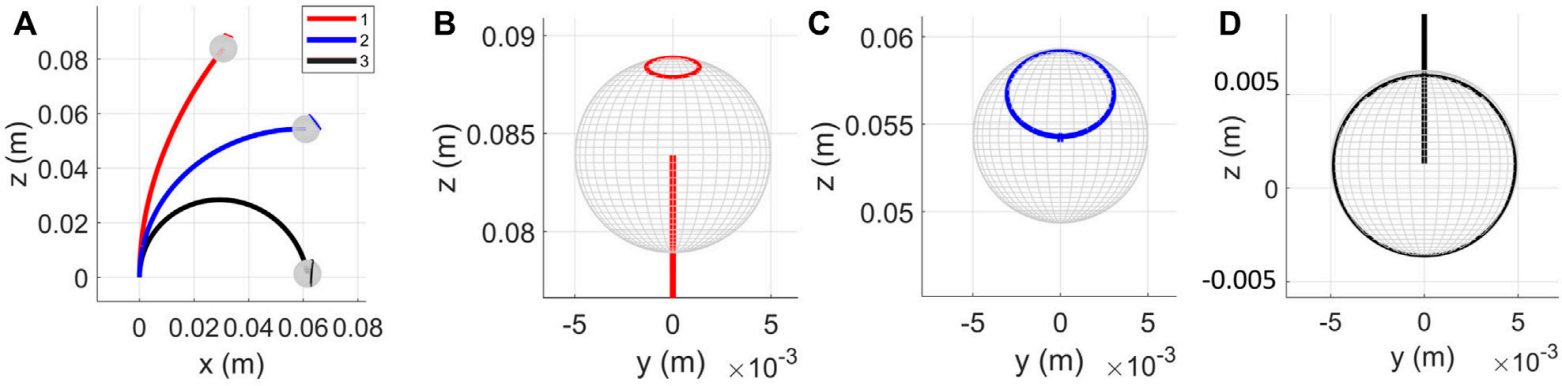
Position redundancy evaluated for three positions for a FAS segment. **(A)** Three configurations considered for evaluation. The service sphere is plotted for configuration one in red **(B)**, two in blue **(C)**, and three in black **(D)**. Friction coefficient is assumed to be zero.

We investigate numerically the FAS redundancy at three positions in its workspace. Making use of the symmetry in the robot workspace, we select three different planar configurations obtained by pulling on the first tendon, shown in Figure 10A. We consider that the frictional and gravitational forces are negligible, an assumption that we relax later on. The one-dimensional curve ***p***_*S*_(*α*) on the service sphere is computed with the continuation process for *α* ∈ [ − *π*, *π*]. For each configuration, the corresponding service spheres have been depicted in Figures 10B–D.

**FIGURE 11:**
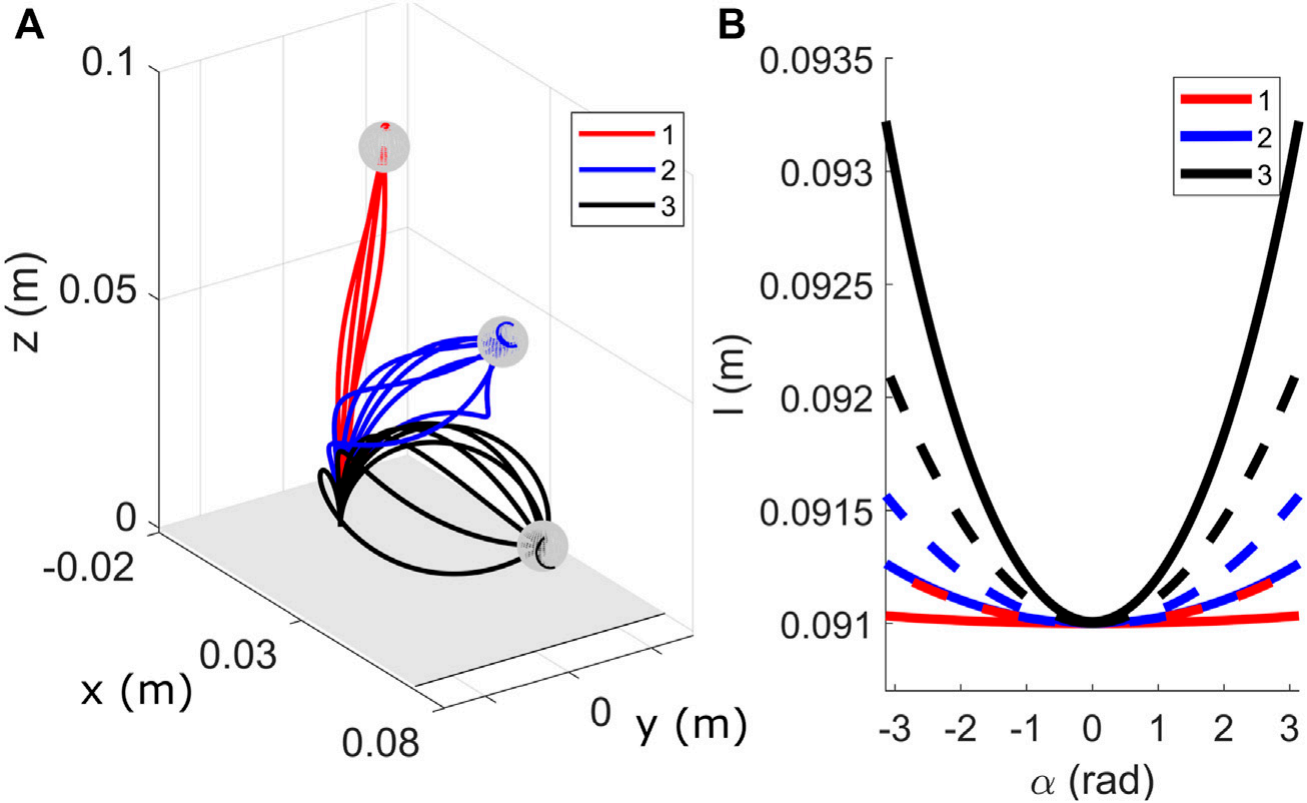
Variation in redundant robot configurations **(A)** Sparse redundant configurations with *μ* = 0.3, for the same tensions considered in Figure 10 **(B)** Plot of variation in length of the backbone with *α*. The dashed lines represent the plots for *μ* = 0.3 and solid lines for *μ* = 0.

We obtain sets of helical robot configurations, as expected, that draw a circular curve on the service sphere. The center of this circle and its radius give indications about the main rotation axis of the segment at the target tip position and the angular displacement achieved around this axis respectively. We observe that the higher the initial curvature of the segment, the larger the disk radius and the angular displacement. We also observe that these large range of orientations are obtained with significant backbone rotations and small backbone translation. The evolution of the segment length with *α* during the continuation process is plotted in Figure 11 for the corresponding three configurations displayed in Figure 10B. We see that the variation in length is in the millimeter range, which aligns with the observations made in the preliminary study. It is interesting to note that these circular curves are not closed-curves. The same point on the service sphere, or a same tip orientation, can be approached using different set of actuation inputs and different robot configurations. Consequently, a specific property of the FAS is to be able to achieve the same tip position and tip tangent with different and discrete values of tip axial rotations, which coincides with the preliminary study.

Finally, we briefly investigate the influence of friction as they have been observed to have a significant effect on the segment shape. The changes of segment length during the backbone rotation and when considering friction are plotted with dashed lines in Figure 11. We observe that the changes in length is larger for configurations one and two when friction is considered to be non-zero compared to the frictionless case, while the opposite trend is observed for configuration 3. The different trends are observed due to the difference in backbone configurations, occurring due to presence of friction.

## 6 Discussion and Perspectives

The FAS design has been shown effective to obtain a TDCR segment with four dof including bending, extension, and twisting. Our initial results show that utilizing twisting as a dof has a beneficial impact on the motion capabilities of a TDCR. To back up our hypothesis, we highlight the capability of the proposed design by qualitatively and quantitatively study its merit through different assessments. The assessments are performed with one of the two physical prototypes and accompanying validations in simulation with the proposed model provide additional insights. In the following, we briefly discuss on the FAS design and then its inherent FTL deployment capability as well as its quantification. Further, we discuss the position redundancy and the static model. Each discussion point is accompanied with promising future directions.

### 6.1 Design of the FAS

All dof can be actuated independently in an extrinsic manner. In addition, our proposed design is simpler than the prototypes presented in Starke et al. (2017) and Gerboni et al. (2015), which involve to combine helical and straight tendons and then may lead to complex tendon routing, especially if several segments are stacked. Special care needs to be taken for the friction between FSD and tendons. On top of that, the use of magnets in type-III FSD introduces new challenges regarding modelling the orientation and position of each FSD. Further, the repulsion forces decrease rapidly as the robot extends, limiting the robot’s maximum length. While the magnetic repulsion force can be improved by increasing the magnet’s thickness, it would in turn limit the robot miniaturization and increase its weight. Among other improvements, finding an alternative to magnets is a future direction to revise the current design.

### 6.2 FTL Deployment Capabilities

We start with a fairly simple deployment strategy, i.e. constant tendon tension and backbone rotation while deploying the backbone. For the preliminary study, the resulting errors are in the range of inaccuracies of the used physical FAS prototype and comparable with the extensible segment TDCR (Amanov et al., 2019, 2016) provided that the deployment length and backbone rotation are small. Note that even in the case of not perfectly distributed FSD along the backbone as can be seen in Figure 7A the resulting errors are sufficiently small. Also note that these are valid postures as this assessment is for FTL use and independent of the model evaluation. In simulation, we show that the deployment strategy needs to be adapted to achieve perfect FTL deployment. The deployment is slightly modified, resulting in a constant tendon tensions with linear change in the backbone rotation and deployment. Indeed, as the backbone must have a constant torsional curvature during the deployment, the angle of the backbone base rotation *α* must be varied linearly w.r.t. the arc-length *s*. While on the one hand this can be seen as a limitation, on the other hand it can be used for FTL deployment strategies in future work.

Another key element to ensure perfect FTL deployment with a TDCR is to include more FSDs. The more FSDs, the closer the TDCR’s tendons to be fully constrained, see Rao et al. (2021) for a discussion on partially and fully constrained tendon paths. In simulation, we have found that perfect FTL deployment can be achieved with 70 FSDs. Also, slight discrepancies between the robot shape and the reference path are observed when 10 FSDs are used.

### 6.3 Area-Based FTL Error

In the literature, FTL deployments are quantified by repeatedly measuring the robot shape during the deployment. In Amanov et al. (2019) and Garriga-Casanovas and Rodriguez y Baena (2018a), a 3D laser scanner is used to obtain a spatial point cloud of the robot. Afterwards, the centerline of the robot’s backbone is extracted relying on a thinning algorithm or on a model of a backbone. Downsides of this labor-intensive approach lie in the use of dedicated hardware and point cloud post-processing.

In contrast, the proposed area-based FTL error (11) is easy to evaluate, interpret, and visualize. The evaluation can be realized with binary images after segmentation and using a simple threshold after combining several binary images as well as summing up all occupied pixels in the respective images. The value can be interpreted as percentage of the normalized surplus of the occupied area during the FTL deployment. Lastly (11) can be visualized as shown in Figure 7. Nevertheless, sources for uncertainty are the manual image segmentation, the low discrete steps, and the dependency of the view. These issues could be alleviated by substituting the manual approach with a learning-based approach enabling an online multi-view evaluation of an FTL deployment. This will be the subject of future work.

Note that an area-based accuracy assessment is used in Chikhaoui et al. (2019) to compare two modeling approaches for a TDCR by projecting a measured point cloud onto several planes. In Girerd et al. (2020), superposition of all intermediate shapes of a CTCR is used to highlight the approximate FTL deployment. A similar approach is utilized in Amanov et al. (2019, 2016), where the overall occupied space over the course of deployment is used to visually show the feasibility. However, none of these qualitative approaches are used to quantify an FTL deployment.

### 6.4 Position Redundancy

Thanks to the four dof, one segment alone already shows interesting properties such as position redundancy as indicated in Figure 9 and Figure 10. Interestingly, the overlaid images shown in Figure 9 and Figure 11 expose that the change in orientation of the end disk is high in comparison to the shape deviation. Note that the segment length has a minor influence on the position redundancy, while *α* and *θ*, respectively, has a major impact as indicated in Figure 9 and Figure 11. The workspace of a TDCR with only two dof, i.e. *κ*_*x*_ and *κ*_*y*_, is a two dimensional manifold. Therefore, a position on this manifold can be reached with different tip orientations, where *α* has a significant contribution. The position redundancy also known as orientability is higher for higher tendon tension as shown in Figure 10. Further, the combined applied tendon tensions are higher for the out-of-plane bending compared to the in-plane bending, see Table 3. Leveraging orientability capability for dynamic obstacle avoidance and for motion planning are promising future research directions.

### 6.5 Static Model of a FAS

During the preliminary study with the physical prototypes, we observed hysteresis behaviours, with velocity-dependent and bending-dependent configurations. We tried to mitigate those phenomena by using multiple measurements via a tactile device to induce noise and reduce measurement biases. The derived static model is calibrated to account for unmodeled effects. It achieves a reasonable accuracy, i.e. average tip error of 4.00 mm corresponding to a relative tip error of 4.39%, compared to 4.10 mm (1.7%) reported for a Cosserat rod-based approach in (Rucker and Webster, 2011). The assumptions of equidistant disk spacing and linear rotation distribution along the backbone are shown to be reasonable approximations. However, they are sources of error for the static model. Specifically modeling the inter-magnetic forces in magnetic spacer disks, and the influence of tendon actuation on the rotation distribution, should be investigated in future work. Additionally, more specific mechanics-based modelling approaches, e.g. (Rucker and Webster, 2011; Chikhaoui et al., 2019), can be adapted for the FAS design and then used to estimate the workspace including position redundancy measures based on (Burgner-Kahrs et al., 2014; Wu et al., 2017) or to extend the FTL deployment strategy proposed by Neumann and Burgner-Kahrs (2016); Amanov et al. (2019) to tendon tension.

In addition to extending the current static model and exploring a dynamic model, developing a kinematic model, which allows to investigate the redundancy, is desirable. This is motivated by an interesting fact stated by Garriga-Casanovas and Rodriguez y Baena (2018b): An extensible TDCR with six dof in the arc space is only capable of five dof at the end effector in the task space due to a loss in rank of the Jacobian matrix for the velocity mapping. Due to the high impact of a geometric twist angle on the orientability, using a FAS might provide a viable solution. Using two concatenated segments provides eight dof in the arc space and could be used to mitigate the above problem.

## 7 Conclusion

We propose a fully actuated segment (FAS) design for tendon-driven continuum robots, which features extensibility and variable tendon routing produced by twist. The actuated twist is implemented by reconsidering spacer disks and leveraging a design of a concentric tube continuum robot. Variable segment lengths are achieved by translating the robot backbone and by using floating spacer disks. Variable tendon routing is achieved by a simple yet effective mechanism to rotate the backbone. As a consequence, the design exploits four dof for one segment; bending in two planes, translation, and rotation. Thanks to the extrinsic actuation, it is a light-weight and slender segment.

The design is qualitatively verified with physical prototypes and further quantitatively validated in simulation with a proposed static model. We demonstrate that a prototype composed of one segment generates helical tendon routing and exhibits position redundancy, which is not possible with previous designs. We also show with a physical prototype and in simulation that it can achieve accurate FTL deployments along planar and complex helical-like paths, the last ones being enabled by the actuated twist. For both paths, we show that the strategy for FTL deployment is fairly simple. Further, we show in simulation the higher the number of spacer disks, the higher the accuracy of an FTL deployment. In addition, we proposed an error measure for FTL deployments and assessed deployment capabilities.

Overall, we prove our hypothesis that utilizing all degrees of freedom has a significant impact on the motion capabilities and, therefore, on the FTL deployment, as well as on position and orientation capabilities. Although our results are preliminary, we believe that the FAS design provides a rich source for future work and has potential in medical and *in situ* inspection applications.

## Data Availability

The raw data supporting the conclusion of this article will be made available by the authors at https://github.com/ContinuumRoboticsLab.
